# Comparison of non-invasive peripheral venous saturations with venous blood co-oximetry

**DOI:** 10.1007/s10877-016-9959-9

**Published:** 2016-11-21

**Authors:** A. M. Belhaj, J. P. Phillips, P. A. Kyriacou, R. M. Langford

**Affiliations:** 1 Southend University Hospital, Prittlewell Chase, Westcliff-on-Sea, Essex SS0 0RY UK; 2 Research Centre for Biomedical Engineering, City, University of London, Northampton Square, London, EC1V 0HB UK; 3 Pain and Anaesthesia Research Centre, St Bartholomew’s Hospital, West Smithfield, London, ECIA 7BE UK

**Keywords:** Venous oxygen saturation, Pulse oximetry, Photoplethysmography, Respiratory-induced intensity variations, Non-invasive monitoring

## Abstract

The estimation of venous oxygen saturations using photoplethysmography (PPG) may be useful as a noninvasive continuous method of detecting changes in regional oxygen supply and demand (e.g. in the splanchnic circulation). The aim of this research was to compare PPG-derived peripheral venous oxygen saturations directly with venous saturation measured from co-oximetry blood samples, to assess the feasibility of non-invasive local venous oxygen saturation. This paper comprises two similar studies: one in healthy spontaneously-breathing volunteers and one in mechanically ventilated anaesthetised patients. In both studies, PPG-derived estimates of peripheral venous oxygen saturations (SxvO_2_) were compared with co-oximetry samples (ScovO_2_) of venous blood from the dorsum of the hand. The results were analysed and correlation between the PPG-derived results and co-oximetry was tested for. In the volunteer subjects,moderate correlation (*r* = 0.81) was seen between SxvO_2_ values and co-oximetry derived venous saturations (ScovO_2_), with a mean (±SD) difference of +5.65 ± 14.3% observed between the two methods. In the anaesthetised patients SxvO_2_ values were only 3.81% lower than SpO_2_ and tended to underestimate venous saturation (mean difference = –2.67 ± 5.89%) while correlating weakly with ScovO_2_ (*r* = 0.10). The results suggest that significant refinement of the technique is needed to sufficiently improve accuracy to produce clinically meaningful measurement of peripheral venous oxygen saturation. In anaesthetised patients the use of the technique may be severely limited by cutaneous arteriovenous shunting.

## Introduction

Photoplethysmography (PPG), a fundamental technology behind the widely used pulse oximeter, is an optical technique that detects periodic blood volume changes in the tissue bed [1]. Pulse oximetry algorithms calculate arterial oxygen saturations (SpO_2_) using the ratio of absorbance of red and infrared light by haemoglobin, exploiting the pulsatile nature of arterial blood to discriminate between arteries and non-pulsatile absorbers in the tissue, such as skin, muscle, bone and pigments [2].

SpO_2_ is by far the most common application of PPG, and while undoubtedly useful, it only gives information about the incoming supply of oxygen. Knowledge of the oxygen saturations in the venous, as well as the arterial blood, reveals more about the balance of oxygen supply and demand in the monitored tissues than the arterial saturations alone. Of the factors affecting the arteriovenous difference in oxygen content (ΔO_2_) across a vascular bed, blood flow through the organ has the greatest influence. Oxygen consumption and haemoglobin concentration also influence ΔO_2_, as demonstrated by the modified Fick equation [3], but in the short term, these tend not to change significantly [4]. This concept is also applicable to the body as a whole, and is the physiological basis of using mixed venous saturations (SvO_2_) as an indication of cardiac output in the assessment of global oxygen delivery. Measurement of SvO_2_, however, requires the placement of a pulmonary artery catheter, which is not without significant risks, and the use of which has seen a steady decline in recent years [5, 6].

**Fig. 1:**
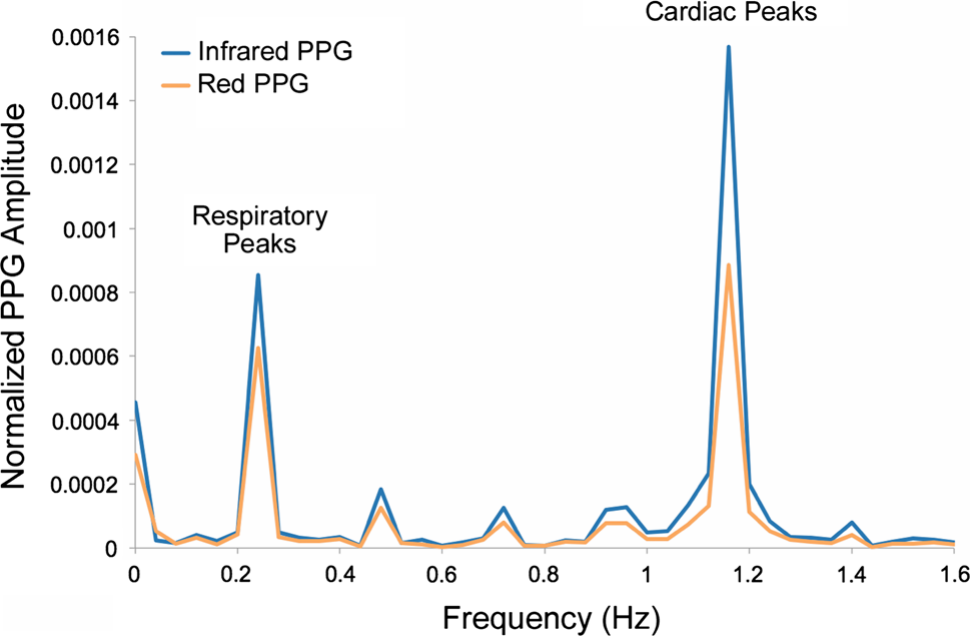
Amplitude spectrum of normalized red and infrared PPG signals recorded from a ventilated anaesthetised patient. Prominent peaks at the respiratory and cardiac frequencies can be seen in both traces

Although peripheral venous saturations cannot replace or infer SvO_2_, direct correlation of central and peripheral venous saturations (SxvO_2_) has been found by Echiadis et al. to be viable in certain cases where the supply or demand balance changes rapidly [4], which would suggest the potential use as continuous non-invasive monitor in critically ill patients. In their study, high frequency artificial venous pulsations, generated by a cuff around the base of the finger were used to calculate SxvO_2_, rather than any intrinsic physiological PPG waveform component. Most interpretations of PPG assume that the only time-varying component of the signal is the variation in volume of the arterial compartment of blood within the tissue [7]. It has been shown that the volume of the veins also varies in response to various physiological and physical effects [8, 9]. Breathing causes periodic variations in blood volume in the peripheral vascular bed, which are evident as Respiratory-Induced Intensity Variations (RIIVs) (see Figs. 1, 4) on recorded PPG signals during spontaneous breathing and mechanical ventilation [10]. Changes in intrathoracic pressure are transmitted from the great veins in the thorax to smaller peripheral veins, and the degree of respiratory modulation depends on central venous pressure (CVP) and intrathoracic pressure [11]. During mechanical ventilation the amplitude of recorded RIIVs are inversely proportional to CVP [12]. Intravenous infusion of fluids has been reported to reduce recorded RIIVs [13], while the removal of blood increases them [14].

Previous research suggests that suitable signal analysis of PPG signals in the respiratory frequency range can produce estimations of SxvO_2_ [15]. Although saturations lower than those of arterial blood have been obtained from analysis of oesophageal [16] and peripheral [17] PPG signals, these estimations have not to date been validated by comparison with co-oximetry of venous blood, considered widely to be the gold standard [18, 19].

The aim of this research was to compare PPG-derived local venous oxygen saturations directly with venous saturation measured from co-oximetry blood samples. The paper describes two separate clinical studies. In the first study, PPG-derived SxvO_2_ was recorded from awake volunteers breathing through a flow resistor to simulate mechanical ventilation and compared to peripheral venous co-oximetry sample values (ScovO_2_) obtained form the dorsal vein of the hand. In the second study, similar measurements were performed in mechanically ventilated anaesthetised patients.

## Methods

### Instrumentation

The measurement system used in both studies consists of a custom made finger pulse oximeter, airway pressure sensor and peripheral venous pressure sensor, attached to the study main monitoring unit.

#### PPG probe

The study pulse oximeter is a standard commercial finger probe shell (GE Datex-Ohmeda^®^, Helsinki, Finland), with the spring clip removed. The shell was fitted with red (660 nm) and infrared (940 nm) light emitting diodes (LEDs) in the upper part of the probe. A photodetector collects the proportion of light not absorbed by tissue on the lower side of the probe. The output feeds into the study monitoring unit where it is amplified using a transimpedance amplifier. A demultiplexer then separates the red (R) and infrared (IR) signals, which are then further separated into alternating current (AC) components, which represent the pulsatile components of the PPG, and direct current (DC) components, which accounts for non-pulsatile tissues in the finger. This separation is achieved using an active filter with pass band ranging from 0.38 to 28.2 Hz. All four signals (AC_R_, DC_R_, AC_IR_ and DC_IR_) are filtered to remove artifact using an active low pass filter of cut-off frequency 16.7 Hz to remove coupled mains and other interference. All filters used in the circuit are based on a second-order Butterworth-type Sallen and Key topology. The signals are digitised using a16-bit data acquisition card (National Instruments Inc., Austin, TX, USA) located within the main monitoring unit and interfaced to a battery-powered notebook computer via a USB cable. All data acquisition functions were performed by a virtual instrument (LabVIEW^®^) on the notebook computer.

#### Airway pressure monitoring

For the volunteer studies, a mouthpiece consisting of a narrow tube (8 mm internal diameter) was used as a flow resistor to allow significant airway pressures to be generated during forced breathing. A port on the mouthpiece was used to measure pressure at the mouth to give an estimation of airway pressure, connected to a signal conditioned pressure sensor (Honeywell Inc., Freeport IL, USA) via an airway gas sampling line. For the studies on mechanically ventilated patients, a short tube with a luer lock gas sampling port was placed between the ventilator tubing and the respiratory filter, and connected to the pressure sensor, as in the volunteer studies.

### Experimental methods

#### Volunteer study

The study on awake volunteers was granted ethical approval by the Senate Research Ethics committee at City University, Northampton Square, London EC1V 0HB, United Kingdom and informed consent was obtained from all participants. ASA Grade 1 and 2 Consenting adults were recruited (9 M, 10 F, mean ± SD age 28.5 ± 7.83 years). Participants with respiratory disease including asthma, vascular disease and clotting disorders were excluded. Subjects were seated in a chair with the right hand resting on a table in front of them. The pulse oximeter probe was placed on the index finger of the right hand. The subjects performed 2 min of voluntary forced inspiration and expiration, in time with a periodic signal, through a narrow tube. These manoeuvres were designed to generate consistent alternating positive and negative airway pressures. The airway pressure, monitored from the mouthpiece, was displayed in real time on a computer screen so that the volunteers could aim for a prescribed rate (12 breaths per minute), rhythm (inspiratory to expiratory time ratio 1:1) and a target airway pressure (+15 to −15 cmH_2_O). Room temperature was controlled at 22 °C and the monitored hand was maintained at the level of the heart. Restrictive clothing which could impede venous return from the arm was removed. At the end of the monitoring period a venous blood sample was taken from the dorsum of the monitored hand and analysed immediately in a Radiometer ABL80 co-oximeter (Radiometer Inc., Copenhagen, Denmark).

#### Ventilated patient study

The study conducted on anaesthetised patients was granted full ethical approval by the East London and City Research Ethics Committee (study reference: 11/LO/0048). Patients requiring general anaesthesia and tracheal intubation were recruited to the study at the Royal London Hospital and written informed consent was obtained from all participants. Measurements were made on 40 ASA Grade 1–3 patients (18 M, 22 F, mean ± SD age 45.8 ± 16.6 years) in the anaesthetic room. Patients with significant respiratory disease, peripheral vascular disease and clotting disorders were excluded. Ambient temperature was in the range 19–23 °C in all cases. After induction of anaesthesia and intubation, patients were ventilated with an airway pressure of 15 cmH_2_O initially, although the pressure was adjusted in a few cases if dictated by clinical requirements. The study monitoring apparatus (finger probe, peripheral venous pressure transducer and gas sampling line) were then attached to the patient. PPG signals and airway pressures were then recorded for 10 min, at the end of which, a venous sample was taken from the dorsum of the hand and analysed using a co-oximeter (Radiometer ABL800) located near the anaesthetic room. The study apparatus was removed before the patient was transferred to the operating theatre.

### Signal processing

Arterial and venous oxygen saturations were calculated from the recorded signals using the normalised AC amplitudes. These are obtained by dividing the AC component of the red and infrared PPG signals (AC_R_ and AC_IR_) by their respective DC signal (DC_R_ and DC_IR_) amplitudes, then converted to a spectral function using a discrete Fourier transform (DFT) producing the amplitude spectra A_R_(*f*) and A_IR_(f) of both signals (see Fig. 1). The amplitudes of the respiratory modulation A_R_(*f*_RESP_) and A_IR_(*f*_RESP_) were found from the heights of the red and infrared spectral peaks in the expected frequency range (0.1–0.4 Hz). The venous oxygen saturation SxvO_2_ was estimated from the ‘ratio-of-ratios’ (enclosed within the parentheses in the equation) using the following relation:

1 $${\text{Sxv}}\text{O}_{2} = 110 - 25\left( {\frac{{A_{R} \left( {f_{RESP} } \right)}}{{A_{IR} \left( {f_{RESP} } \right)}}} \right)$$

This equation is based on the often-quoted equation used to calibrate Nellcor commercial pulse oximeters; a linear approximation of empirical data obtained from volunteer studies [20].

### Statistical analysis

Outliers were excluded using a rejection threshold defined by Grubbs’ test [21] (Thompson tau test) with a 95% confidence level. For both studies, the root mean squared error (RMSE) for the venous saturations calculated from the PPG (${\text{SxvO}}_{2}$) signals versus co-oximetry derived venous saturation (${\text{ScovO}}_{2}$) using:

2 $$RMSE = \sqrt {\frac{{\sum\nolimits_{i = 1}^{n} {({\text{SxvO}}_{2} - {\text{ScovO}}_{2} )^{2} } }}{n}}$$

Paired *t*-tests were performed on all arterial and venous calculated saturation datasets to determine whether there was a statistically significant difference between SxvO_2_ and SpO_2_, and between SxvO_2_ and ScovO_2_. The limits of agreement between SxvO_2_ and ScovO_2_ were obtained from the Modified Bland–Altman plot [22], whereby the difference between SxvO_2_ and ScovO_2_ were plotted against the established gold standard (ScovO_2_).

## Results

### Volunteer study

**Fig. 2:**
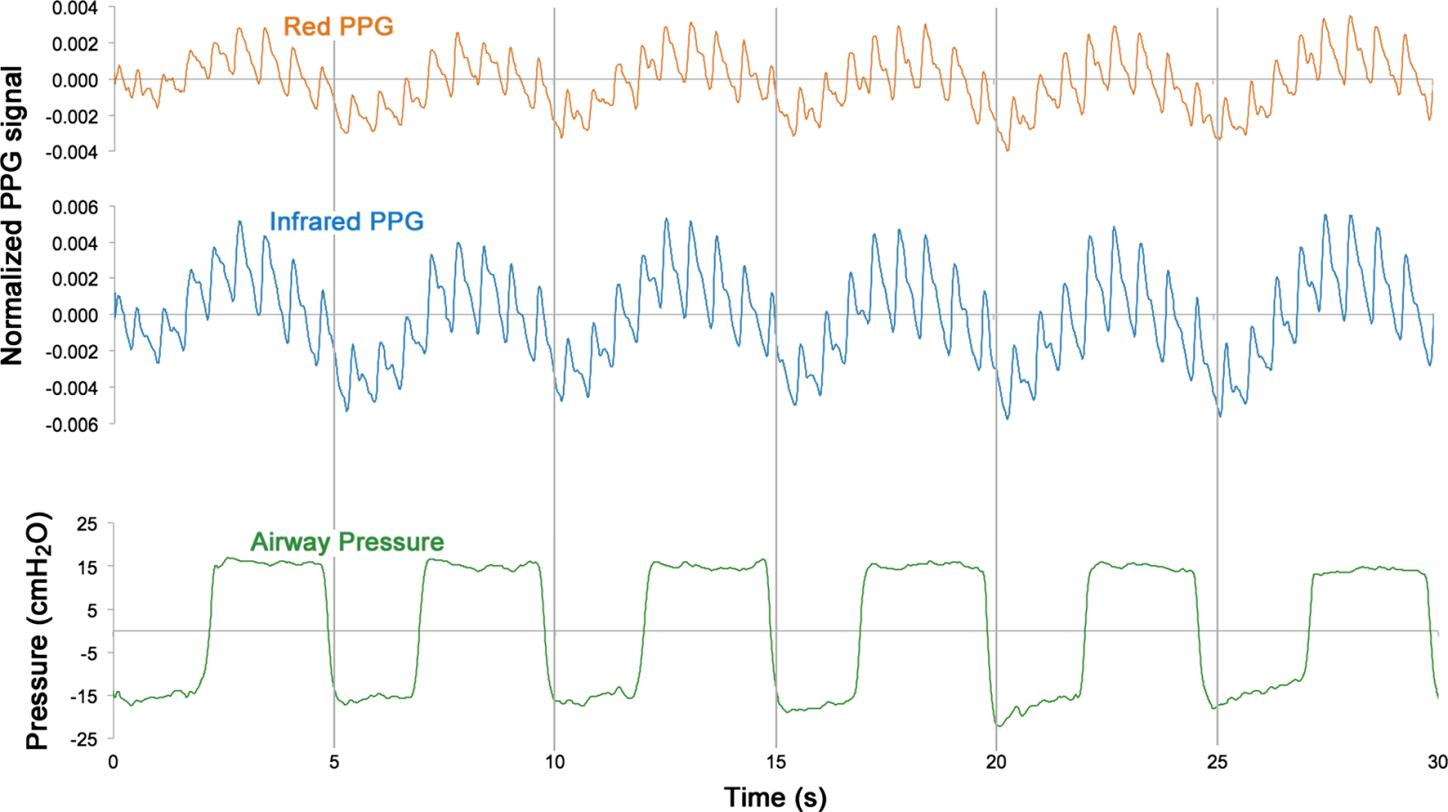
30 s sample of red and infrared photoplethysmographic signals with simultaneously recorded airway pressures from one volunteer subject during timed forced breathing manoeuvre

The respiratory modulations of the PPG signal were observed clearly in all subjects, with maximum attenuation of light occurring during the expiratory phase. Figure 2 shows a 60 s sample from a typical subject of the normalised red (AC_R_/DC_R_) and infrared (AC_IR_/DC_IR_) PPG signals with airway pressure simultaneously recorded during the forced breathing manoeuvres.

**Fig. 3:**
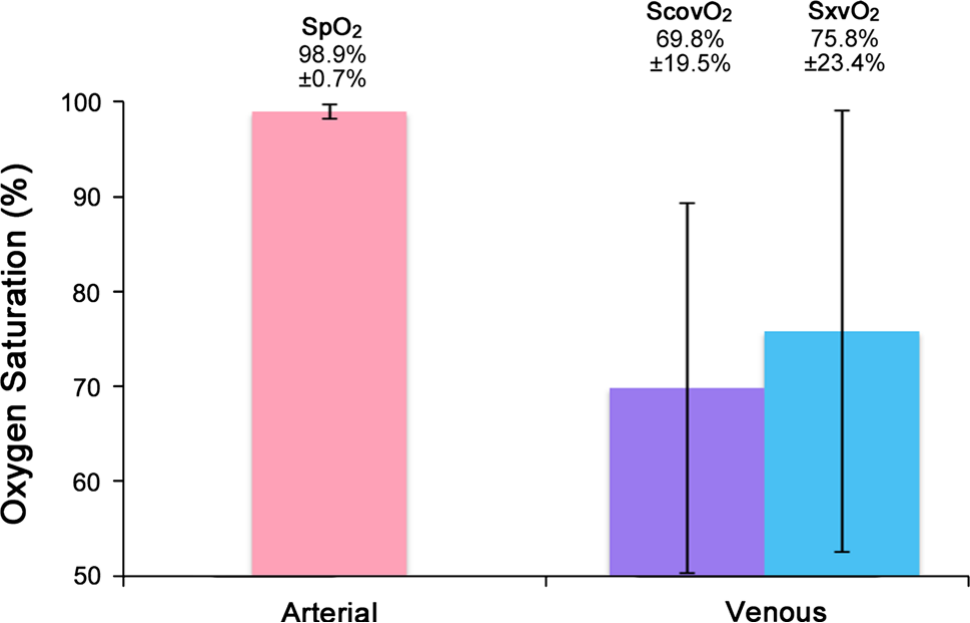
Barchart showing mean (±SD) arterial oxygen saturation (from pulse oximeter) and venous oxygen saturations from co-oximetry (ScovO_2_)and PPG-derived (SxvO_2_) method recorded in awake volunteers (*n* = 17)

Nineteen volunteers were recruited to this study. Two results were identified as outliers and removed from the dataset. Figure 3 shows the mean arterial saturation (SpO_2_) and the mean venous saturations (ScovO_2_ and SxvO_2_) for all subjects. The mean difference between PPG derived peripheral venous saturations (SxvO_2_) and arterial saturations as measured by the commercial pulse oximeter (SpO_2_) was −23.4% (*P* < 0.05, *n* = 17). The experimental method overestimated the venous oxygen saturation; the mean (±SD) difference between between SxvO_2_ values and co-oximetry derived venous saturations (ScovO_2_) was +5.65 ± 14.3%.

**Fig. 4:**
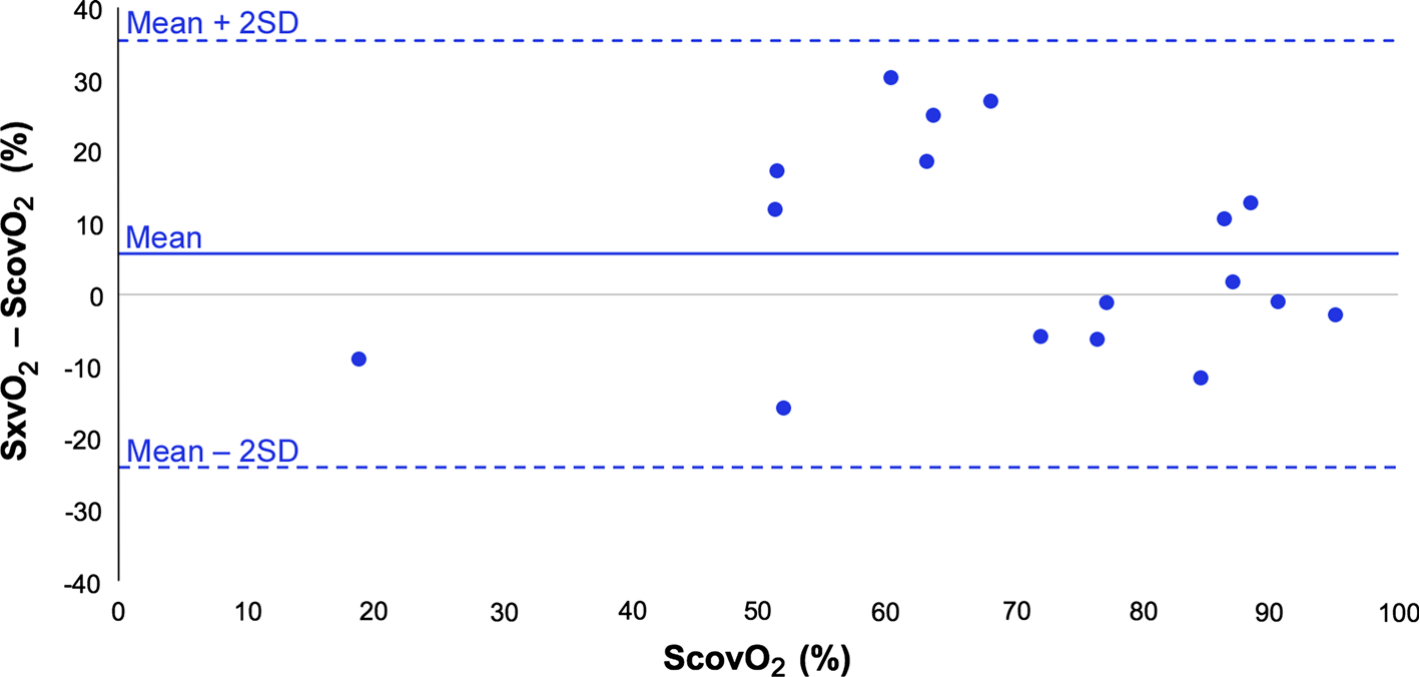
Modified Bland–Altman plot showing mean difference in venous oxygen saturation between PPG-derived method (SxvO_2_) and reference method (co-oximetry ScovO_2_) in awake volunteers

Figure 4 shows a modified Bland–Altman plot of the difference between PPG-derived peripheral venous saturation (SxvO_2_) and co-oximetry values (ScovO_2_) against the gold standard, ScovO_2_. The results show moderate correlation (*r* = 0.81, RMSE = 15.1%, *P* = 0.137) and wide limits of agreement between the two methods of measurement.

### Ventilated patient study

**Fig. 5:**
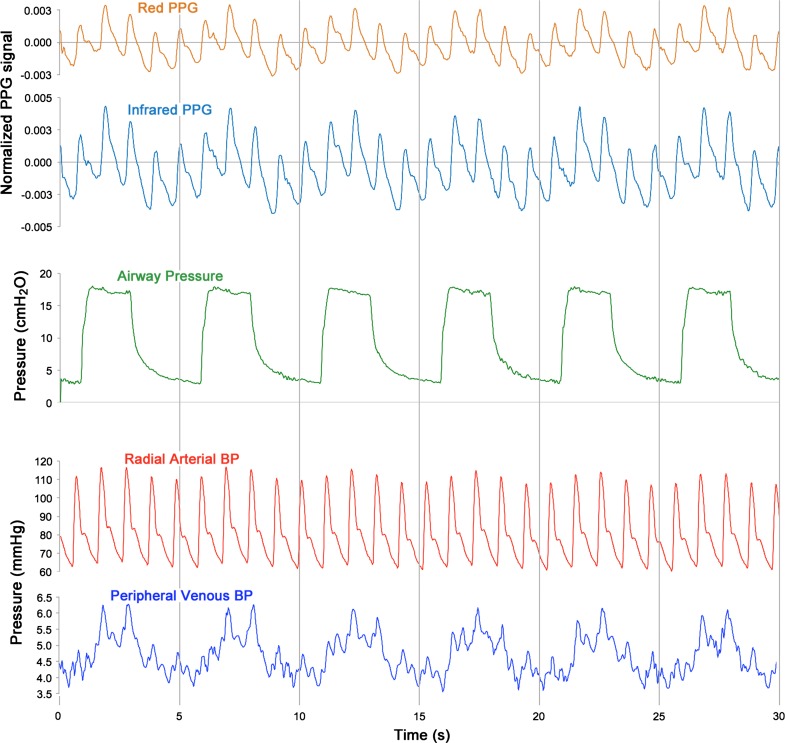
30 s sample of red and infrared photoplethysmographic signals with simultaneously recorded airway pressures, radial arterial blood pressures and peripheral venous blood pressures from one ventilated anaesthetised patient

**Fig. 6:**
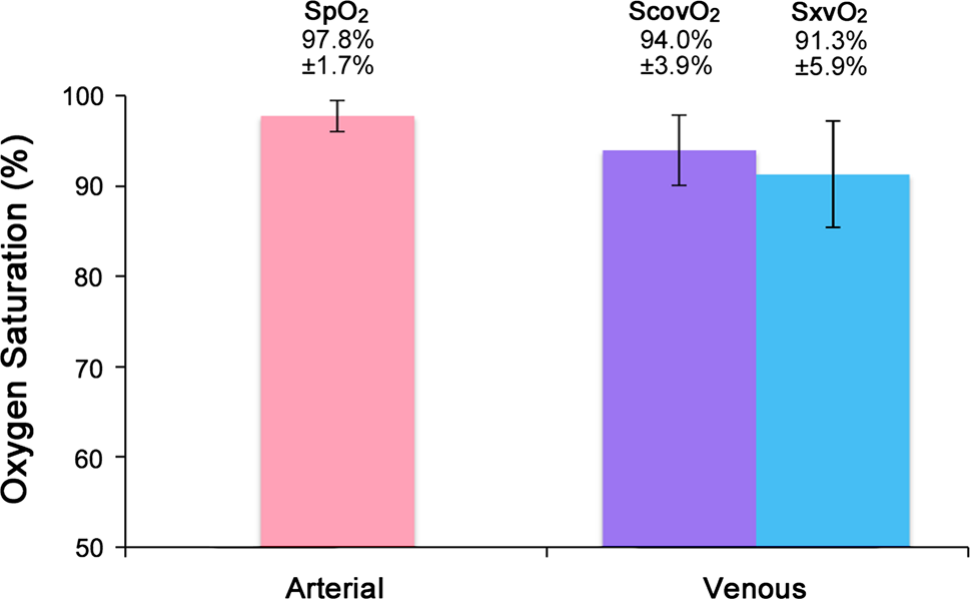
Barchart showing mean (±SD) arterial oxygen saturation (from pulse oximeter) and venous oxygen saturations (from co-oximetry and PPG-derived method) recorded in ventilated patients (*n* = 38)

Forty subjects were recruited in this study. Figure 5 shows a 60 s sample from a typical subject of the normalised red (AC_R_/DC_R_) and infrared (AC_IR_/DC_IR_) PPG signals with simultaneous airway pressure and peripheral venous pressure waveforms. As with the volunteer study, significant modulation of the PPG signal was observed in time with ventilatory variations in airway pressure and peripheral venous pressure. Two results were identified as outliers and removed from the dataset. Figure 6 shows the mean arterial saturation (SpO_2_) and the mean venous saturations (ScovO_2_ and SxvO_2_) for all subjects. The mean difference between the PPG-derived peripheral venous saturations (SxvO_2_) and the arterial saturation as measured by the commercial pulse oximeter (SpO_2_) was −3.81% (*P* < 0.05, *n* = 38). The experimental method underestimated the venous oxygen saturation; a mean (±SD) difference of −2.67%, ±5.89% between SxvO_2_ and the ScovO_2_ was observed. It should be noted that the blood sampled from the hands of the anaesthetised patients (ScovO_2_) showed considerably higher mean oxygen saturation than was seen in the volunteer subjects (94.0% in patients compared with 69.9% in volunteers).

**Fig. 7:**
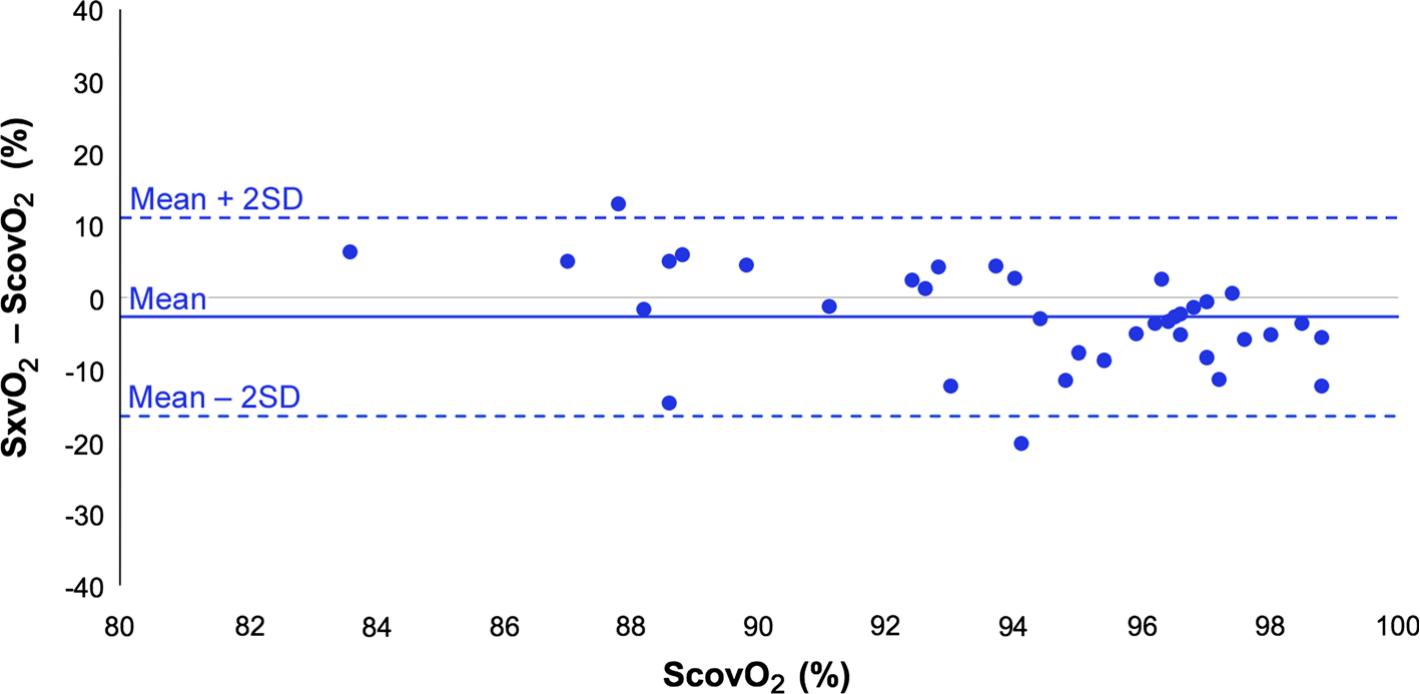
Modified Bland–Altman plot showing mean difference in venous oxygen saturation between PPG-derived method (SxvO_2_) and reference method (co-oximetry ScovO_2_) in ventilated patients

A modified Bland–Altman plot of the difference between PPG-derived peripheral venous saturation (SxvO_2_) and co-oximetry samples (ScovO_2_) plotted against ScovO_2_ (Fig. 7), showed considerable inter-subject variability and wide limits of agreement This variability combined with the narrow range of reference venous saturations investigated (83.6–98.8%) produced extremely poor correlation and a significant difference between the two methods (*r* = 0.10, RMSE = 6.19%, *P* < 0.05).

## Discussion

In the volunteer group, venous oxygen saturations that were significantly lower than those of arterial blood were calculated from PPG analysis in the respiratory frequency range (SxvO_2_), and moderate correlation was found between these values and those obtained by co-oximetry (ScovO_2_). Note that a normally-breathing control group was not included as the respiratory component of the PPG is difficult to resolve under such circumstances. The effect of the airway pressure itself was the subject of an earlier volunteer study using a similar experimental setup [23] which concluded that forced breathing caused a fall in reported arterial oxygen saturation of 1.9% compared to control measurements. SxvO_2_ values in the anaesthetised subjects were also found to be significantly lower than arterial saturations (SpO_2_), but in contrast to the volunteer subjects, SxvO_2_ values were high and correlated poorly with co-oximetry. This may be largely attributed to cutaneous arteriovenous (AV) shunt induced by anaesthesia [24].

The circulation of the skin has a highly specialised vascular arrangement. At rest and in cool ambient temperature, a state of relative vasoconstriction prevails, controlled by the sympathetic innervation. Blood flow can be greatly increased through vasodilation, under the central control of the hypothalamus, in conditions where extensive heat transfer is required [25]. The arteries supplying the skin are located deep in the hypodermis and give rise to plexuses of anastomosing vessels. Numerous shunts provide direct AV communications which play an important role in thermoregulation, and are densely innervated with sympathetic fibres [26]. In addition, the dermis in the finger tips, from which PPG is recorded, contains highly convoluted AV anastomoses known as glomus bodies, providing more direct AV mixing [27].

When thermal or pharmacological vasodilation occurs, the oxygen content of peripheral venous blood rises significantly. In previous work studying the arterialisation of venous blood in the heated hand, venous blood gas values were found to be consistently in the arterial range [28]. Intravenous anaesthetics, volatile agents and opioids, all of which were administered to the study subjects, cause impairment of the hypothalamic response to hypothermia and direct vasodilator action causing the skin to appear flushed and veins to become prominent. The extensive AV mixing is likely to have resulted in the high oxygen saturations observed in the dorsal vein blood samples (ScovO_2_) in the anaesthetised subjects and may account for the poor correlation between SxvO_2_ and ScovO_2_. The PPG-derived SxvO_2_ estimates were significantly lower than ScovO_2_ in this group, which suggests that arterialisation of dorsal vein blood is occurring.

A second limitation of the anaesthetised patients study was highlighted by observation of very high peripheral venous oxygen saturation values. The administration of high flow oxygen to patients prior to anaesthesia, which continued after anaesthetic induction to allow for the full action of muscle relaxants before tracheal intubation, probably results in the tissue beds being flooded with oxygen resulting in tissue hyperoxia and resulting venous hyperoxaemia. Although the fraction of inspired oxygen (F_i_O_2_) was reduced to 0.3 as soon as the airway was secured to minimise the effect of preoxygenation, the short period of monitoring in the anaesthetic room before transfer into the operating theatre did not allow enough time for return of arterial oxygen tension to normal values. This limitation could perhaps be overcome in future studies by recording PPG during or after the surgical procedure, which would provide time for washout of excess oxygen, thus allowing for a meaningful difference in arterial and venous oxygen saturations.

In the volunteer group, room temperature was controlled, and although this does not eliminate individual differences in cutaneous vascular tone and AV shunting, it has been possible to obtain consistently low SxvO_2_ that correlated well with the ScovO_2_ values. There was, however, wide spread of data, which may be attributed to intersubject variability in technique of forced breathing. The calculation of SxvO_2_ depends on the ratio of the signal amplitude between the red and infrared peaks in the respiratory frequency range. Inconsistent breathing technique causes the respiratory frequency to become indeterminate, causing errors in the calculated amplitudes of the respiratory red and infrared signals at the respiratory frequency. This affects the ratio-of-ratios and thus the calculated saturations.

The finger is often chosen as a convenient monitoring site for studying PPG, and indeed for routine clinical monitoring, but it is clear from these studies and previous work that further research is needed to enhance our understanding of the behaviour of the venous and arterial compartments within the tissues of the finger and the effect of this behaviour on pulse oximetry signals. Furthermore the effect of other physiological factors such as cutaneous arteriovenous shunt and how these are affected by physical variables such as ambient temperature warrant further study.

In conclusion, these results suggest that measurement of peripheral venous oxygen saturation remains a challenge. Although there is growing interest in utilising this concept in the monitoring of regional circulations, refinement of the technique, including application of more suitable algorithms and calibration functions, are perhaps needed to sufficiently improve accuracy to produce clinically meaningful results from peripheral sites. Cutaneous arteriovenous shunt induced by anaesthesia may especially limit the use of this technique in anaesthetised patients. Application of suitable PPG sensors [15] to the splanchnic circulation however, where AV anastomoses do not interfere with blood flow, could provide valuable clinical tools for assessment of oxygen uptake and metabolic status of vital organs.

## References

[1] Allen J (2007). Photoplethysmography and its application in clinical physiological measurement. Physiol Meas.

[2] Moyle JTB (2002). Pulse oximetry.

[3] Stock MC, Ryan ME (1996). Oxygen consumption calculated from the Fick equation has limited utility. Crit Care Med.

[4] Echiadis AS, Crabtree VP, Bence J, Hadjinikolaou L, Alexiou C, Spyt TJ, Hu S (2007). Non-invasive measurement of peripheral venous oxygen saturation using a new venous oximetry method: evaluation during bypass in heart surgery. Physiol Meas.

[5] Wiener RS, Welch HG (2007). Trends in the use of the pulmonary artery catheter in the United States, 1993–2004. J Am Med Assoc.

[6] Harvey S, Harrison DA, Singer M, Ashcroft J, Jones CM, Elbourne D, Brampton W, Williams D, Young D, Rowan K (2005). Assessment of the clinical effectiveness of pulmonary artery catheters in management of patients in intensive care (PAC-Man): a randomised controlled trial. Lancet.

[7] Phillips JP, Belhaj A, Shafqat K, Langford RM, Shelley KH, Kyriacou PA. Modulation of finger photoplethysmographic traces during forced respiration: venous blood in motion? In: Conference proceedings of the IEEE engineering in medicine and biology society; 2012. p. 3644–7. 10.1109/EMBC.2012.6346756 23366717

[8] Nilsson L, Johansson A, Kalman S (2003). Respiratory variations in the reflection mode photoplethysmographic signal. Relationships to peripheral venous pressure. Med Biol Eng Comput.

[9] Phillips JP, Belhaj A, Langford RM, Kyriacou PA. Effect of respiratory-induced intensity variations on finger SpO_2_ measurements in volunteers. In: Conference proceedings of the IEEE engineering in medicine and biology society; 2013. p. 3937–40. 10.1109/EMBC.2013.6610406 24110593

[10] Shelley KH (2007). Photoplethysmography: beyond the calculation of arterial oxygen saturation and heart rate. Anesth Analg.

[11] Dorlas JC, Nijboer JA (1985). Photo-electric plethysmography as a monitoring device in anaesthesia: application and interpretation. Br J Anaesth.

[12] Partridge BL (1987). Use of pulse oximetry as a noninvasive indicator of intravascular volume status. J Clin Monit.

[13] Cannesson M, Attof Y, Rosamel P, Desebbe O, Joseph P, Metton O, Bastien O, Lehot JJ (2007). Respiratory variations in pulse oximetry plethysmographic waveform amplitude to predict fluid responsiveness in the operating room. Anesthesiology.

[14] Gesquiere MJ, Awad AA, Silverman DG, Stout RG, Jablonka DH, Silverman TJ, Shelley KH (2007). Impact of withdrawal of 450 ml of blood on respiration-induced oscillations of the ear plethysmographic waveform. J Clin Monit Comput.

[15] Phillips JP, Kyriacou PA, Jones DP, Shelley KH, Langford RM (2008). Pulse oximetry and photoplethysmographic waveform analysis of the esophagus and bowel. Curr Opin Anesthesiol.

[16] Walton ZD, Kyriacou PA, Silverman DG, Shelley KH (2010). Measuring venous oxygenation using the photoplethysmograph waveform. J Clin Monit Comput.

[17] Thiele RH, Tucker-Schwartz JM, Lu Y, Gillies GT, Durieux ME (2011). Transcutaneous regional venous oximetry: a feasibility study. Anesth Analg.

[18] Shapiro BA, Peruzzi WT, Kozelowski-Templin R (1994). Clinical application of blood gases.

[19] Webster JG (1997). Design of pulse oximeters.

[20] Rusch TL, Sankar R, Scharf JE (1996). Signal processing methods for pulse oximetry. Comput Biol Med.

[21] Grubbs FE (1969). Procedures for detecting outlying observations in samples. Technometrics.

[22] Krouwer JS (2008). Why Bland–Altman plots should use X, not (Y + X)/2 when X is a reference method. Stat Methods.

[23] Phillips JP, Belhaj AM, Langford RM, Kyriacou PA, Effect of respiratory-induced intensity variations on finger SpO_2_ measurements in volunteers. In: Conference proceedings of the IEEE engineering in medicine and biology society; 2013. p. 3937–40. 10.1109/EMBC.2013.6610406 24110593

[24] Kim JM (1986). Pulse oximetry and circulatory kinetics associated with pulse volume amplitude measured by photoelectric plethysmography. Anesth Analg.

[25] Rhoades R, Bell DR (2013). Medical physiology: principles for clinical medicine.

[26] Boron WF, Boulpaep EL (2012). Medical physiology: a cellular and molecular approach.

[27] Young B, Heath JW, Burkitt HG, Wheater PR (2000). Wheater’s functional histology: a text and colour atlas.

[28] Zello GA, Smith JM, Pencharz PB, Ball RO (1990). Development of a heating device for sampling arterialised venous blood from a hand vein. Ann Clin Biochem.

